# A Recurrent *BRCA2* Mutation Explains the Majority of Hereditary Breast and Ovarian Cancer Syndrome Cases in Puerto Rico

**DOI:** 10.3390/cancers10110419

**Published:** 2018-11-02

**Authors:** Hector J. Diaz-Zabala, Ana P. Ortiz, Lisa Garland, Kristine Jones, Cynthia M. Perez, Edna Mora, Nelly Arroyo, Taras K. Oleksyk, Miguel Echenique, Jaime L. Matta, Michael Dean, Julie Dutil

**Affiliations:** 1 Cancer Biology Division, Ponce Research Institute, Ponce Health Sciences University, Ponce, Ponce, PR 00716-2348, USA; diazhectorjoel@gmail.com (H.J.D.-Z.); narroyo@psm.edu (N.A.); jmatta@psm.edu (J.L.M.); 2 Cancer Control and Population Sciences Program, Comprehensive Cancer Center, University of Puerto Rico, San Juan, PR 00936-5067, USA; ana.ortiz7@upr.edu; 3 Cancer Genomics Research Laboratory, Leidos Biomedical Research, Inc., Frederick, MD 21702, USA; garlandlr@mail.nih.gov (L.G.); kristine.jones@nih.gov (K.J.); 4 Department of Biostatistics and Epidemiology, Graduate School of Public Health, University of Puerto Rico, San Juan, PR 00936-5067, USA; cynthia.perez1@upr.edu; 5 Department of Surgery, School of Medicine, University of Puerto Rico and University of Puerto Rico Comprehensive Cancer Center, San Juan, PR 00936-5067, USA; emora@cccupr.org; 6 Biology Department, Oakland University, Rochester, MI 48309-4454, USA; oleksyk@oakland.edu; 7 Department of Biology, University of Puerto Rico in Mayaguez, Mayaguez, PR 00681, USA; 8 Cancer Center, Auxilio Mutuo Hospital, San Juan, PR 00936-5067, USA; echeniquemm@gmail.com; 9 Laboratory of Translational Genomics, Division of Cancer Epidemiology and Genetics, National Cancer Institute, Gaithersburg, MD 20877, USA; deanm@mail.nih.gov

**Keywords:** breast cancer genetics, *BRCA1*/*BRCA2*, founder effect, Hispanic and Latino populations

## Abstract

Breast cancer is the most common cause of cancer diagnosis in women and is responsible for considerable mortality among the women of Puerto Rico. However, there are few studies in Puerto Rico on the genetic factors influencing risk. To determine the contribution of pathogenic mutations in *BRCA1* and *BRCA2*, we sequenced these genes in 302 cases from two separate medical centers, who were not selected for age of onset or family history. We identified nine cases that are carriers of pathogenic germline mutation. This represents 2.9% of unselected cases and 5.6% of women meeting National Comprehensive Cancer Network (NCCN) criteria for BRCA testing. All of the identified pathogenic mutations were in the *BRCA2* gene and the most common mutation is the p.Glu1308Ter (E1308X) mutation in *BRCA2* found in eight out of nine cases, representing 89% of the pathogenic carriers. The E1308X mutation has been identified in breast and ovarian cancer families in Spain, and analysis of flanking DNA polymorphisms shows that all E1308X carriers occur on the same haplotype. This is consistent with *BRCA2* E1308X being a founder mutation for the Puerto Rican population. These results will contribute to better inform genetic screening and counseling of breast and ovarian cancer cases in Puerto Rico and Puerto Rican populations in mainland United States.

## 1. Introduction

Pathogenic variants in the highly penetrant susceptibility genes *BRCA1* and *BRCA2* confer an increased lifetime risk of breast, ovarian and other cancers [1,2]. For women at high risk of developing breast cancer (BC), risk reduction options include increased surveillance [3], chemoprevention [4,5] and prophylactic surgery [6,7]. Research has shown that women who are educated about their increased risk of breast cancer are more likely to engage in risk-reducing behaviors and early detection strategies such as monthly self-breast exam, physician visits, mammography and breast magnetic resonance imaging (MRI) screening [8]. In newly diagnosed cancer patients, BRCA testing also reportedly affects surgical decision-making [8,9,10]. BRCA testing in family relatives of identified carriers can discriminate between those at high risk and spare others from unnecessary risk-reducing options [8].

While most populations of Latin America and the Caribbean result from the admixture of ancestors from African, European and Native American origins, substantial heterogeneity has been observed within and across countries [11]. Such variability translates into distinct genetic architecture underlying diseases with a hereditary component. The prevalence of hereditary cancers attributed to pathogenic variants in the *BRCA1* and *BRCA2* genes in unselected BC patients from Latin America and the Caribbean varies from 1.2% in Columbia to 27.1% in the Bahamas [12]. As a result of founder effects, some populations have shown decreased genetic variability in the spectrum of *BRCA* mutations observed, but others such as Argentina exhibit considerable diversity with fewer recurrent mutations [12]. 

In Puerto Rico, our previous work indicates that a few recurrent mutations may explain the majority of hereditary cancer cases [13]. However, this preliminary work relied on a limited sample size. The objective of the current study was to estimate the frequency of the *BRCA* mutations in a group of breast cancer patients unselected for age of onset or family history of cancer and identify the common pathogenic variants in this population. Understanding the genetic basis of hereditary cancers in a given population is essential for targeting screening, prevention strategies and clinical management of cancers that incorporate the unique features characterizing each population. 

## 2. Results

### 2.1. Characteristics of the Study Population

The description of demographic, hormonal and clinical characteristics of the study population is presented in Table S1. All study participants had been previously diagnosed with BC but were not selected for age of onset or family history of breast or other cancers. Most of the study population was married (53.2%) and of diverse socioeconomic status as indicated by a roughly equal proportion of participants with an education level of up to high school (40.2%) and holding at least a bachelor’s degree (37.1%). In terms of hormonal and pregnancy history, most women had a menarche before 13 years of age (56.9%), had a history of pregnancy (88.8%) with one to two children (44.7%), and were currently undergoing or had undergone menopause (67.2%). Approximately forty percent had had their breast cancer diagnosis at or before the age of 50 years. The most common tumors were ductal invasive carcinomas (71.1%), smaller than 2 cm (64.4%) and negative for lymph node invasion (67.7%). Overall, family history was reported as follows: 33.7% had family history of breast cancer in at least one relative, 3.1% of ovarian cancer, 1.7% of male BC, 18.8% of other BRCA-associated cancers (prostate, pancreas and melanoma). Based on personal and familial history of cancer, 45.9% of the women enrolled met the National Comprehensive Cancer Network (NCCN) criteria for BRCA genetic testing (version 2.2017) [14]. Breast tumor and family history characteristics of the study population are presented in Table S2. Patients were enrolled from two major urban centers (San Juan in the North East and Ponce in the South), and their municipality of residence represented 53 of the 78 municipalities of Puerto Rico (Figure S1).

### 2.2. BRCA1 Variants

A total of 64 variants were identified in the *BRCA1* gene, 48 of which are present at a frequency of less than 1% in the 1000 Genomes PUR population (Puerto Ricans from Puerto Rico) or have not been reported in this population. Among those, there were the two positive controls from the Coriell Biorepository: NA13715 *BRCA1* 5382insC, NA14638 *BRCA1* IVS5-11T>G. No additional known pathogenic variants were identified in this cohort (Table 1). In addition, six missense variants (Table 2) and four intronic variants (Table S3) were classified as variants of unknown significance (VUS) or had conflicting reports of pathogenicity in ClinVar [15]. The remaining variants are classified as benign or likely benign and are not reported.

### 2.3. BRCA2 Variants

Out of the 102 variants identified in the *BRCA2* gene, 79 had a reported frequency of less than 1% or were absent in the 1000 Genomes PUR sample. Two pathogenic variants (Table 1) were observed: a deletion of five base pairs resulting in a frameshift and a stop at position 599 of the *BRCA2* protein (c.1794_1798delATTTT); and a nonsense mutation resulting in the change of a glutamate for a stop at position 1308 (c.3922G>T, rs80358638). Interestingly, the latter was recurrent in eight unrelated individuals from this study population. There were an additional eight missense variants (Table 2) and seven intronic variants (Table S3) with conflicting reports of pathogenicity in ClinVar or classified as VUS. Variants classified as benign or likely benign are not reported.

### 2.4. Prediction of Functionality of Missense Variants of Uncertain Significance

To evaluate the likelihood of pathogenicity of VUS, rare missense variants were submitted to in silico prediction models including Align-GVGD [17], Breast Cancer Gene Prior Probabilities of the Huntsman Cancer Institute (HCI BrCa) [18], Polyphen2 [19], SIFT [20] and Provean [21,22] (Table 3). In *BRCA1*, the H1421Y variant obtained a score of C15 on align GVGD and was predicted possibly damaging and damaging by Polyphen and SIFT. In *BRCA2*, K2950N had the highest GVGD score (C35) and was predicted to impact the protein function by three of the models tested (HCI BrCa, Polyphen2, and SIFT). The majority of the remaining missense variants of uncertain significance were predicted benign by most or all models.

### 2.5. BRCA Mutation Prevalence and Clinical Characteristics of the Carriers

A total of nine women were found to carry a known pathogenic mutation, which corresponds to a prevalence of 2.9% (95% confidence interval (CI) 1.5–5.5%) in this population of BC patients unselected for age of onset or family history. The proportion of *BRCA* carriers in BC cases diagnosed at or before 50 years of age was 5.2% (CI 2.4–10.8%). When only patients that meet the NCCN criteria for BRCA testing [14] are selected (45.9% of the cases), the prevalence increases to 5.6% (CI 2.8–11.2%). Interestingly, *BRCA2* mutations represented 100% of the mutation carriers. Overall, carriers showed considerable variability in tumor pathology (Table 4). There was no clear bias in tumor type, site, size and characteristics of the cell surface receptors (Estrogen ER, Progesterone PR or Human epithelial growth factor, HER2 receptors). The family history characteristics of the carriers did not stand out with the exception of a stronger family history for male breast cancer, reported in second degree relatives of three *BRCA2* E13908X carriers (Table 4). It is noteworthy that seven of the nine carriers met the NCCN criteria for *BRCA* testing. For the remaining two, insufficient data was available to determine whether there was a personal or family history of breast cancer sufficient to justify genetic testing. The patient UPR1024, *BRCA2* c.1794_1798del5 carrier, was diagnosed at 52 years of age and did not report any family history of cancer. This individual would have been recommended for genetic testing only if triple negative (ER-, PR- and HER2-negative). PRI739, a *BRCA2* E1308X carrier, was diagnosed at 53 and two first-degree relatives with a history of prostate cancer: his father was diagnosed at 70 years and a brother who died at 47 years. Pathologies from those cancers were not available to determine whether the Gleason scores were ≥7, in which case this patient would have met the criteria for *BRCA* testing.

### 2.6. Genomic Context in BRCA2 E1308X Carriers

**Figure 1:**
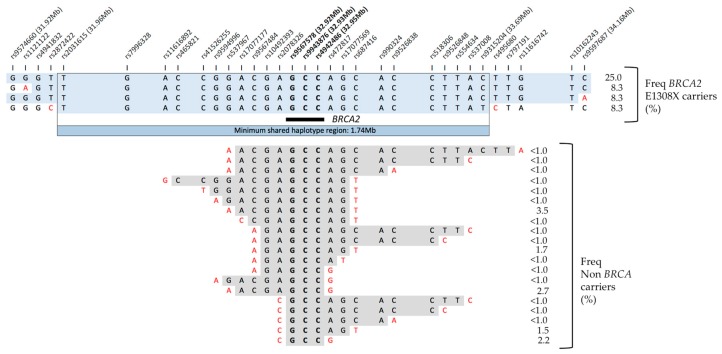
Shared haplotype and frequencies in a 2.24 Megabase (Mb) region around the *BRCA2* gene in carriers of the E1308X pathogenic variant (*n* = 6) and non-BRCA carriers (*n* = 201). A total of 73 markers were phased in a 2.6 Mb region, but only 33 are represented. For non-BRCA carriers, only a fraction of the observed haplotypes are illustrated, representing the haplotypes that match those of the carriers within the *BRCA2* gene.

Given the high number of carriers of the *BRCA2* E1308X mutation, haplotype analysis was conducted to assess whether the mutation has a common ancestral origin (Figure 1). For a subset of 207 study participants, including six of the eight *BRCA2* E1308X carriers, genome-wide SNP array data was available to determine the haplotype in a 6 Mb region around *BRCA2*. All six genotyped carriers of the *BRCA2* E1308X pathogenic variant shared a common haplotype in a region spanning at least 1.74 Mb. This haplotype was absent in 199 non-carriers. Identity by state (IBS) analysis between all pairs of individuals using linkage disequilibrium pruned genome-wide markers confirmed that these carriers were not closely unrelated (identity by descent (IBD) PI-HAT for carrier pairs < 0.125). There was no obvious bias in the global ancestry proportions of *BRCA2* E1308X carriers, although none of the carriers were within the upper end of the African ancestry distribution (Figure S2). All examined *BRCA2* E1308X carriers shared at least one chromosome of European origin in the *BRCA2* region (Figure S2).

## 3. Discussion

We have characterized the *BRCA1* and *BRCA2* variants in 307 unselected breast cancer patients in Puerto Rico. The only published study on the *BRCA* mutation spectrum in Puerto Rico analyzed 23 high-risk patients and identified six unique mutations in 12 carriers. In total, seven unique pathogenic variants have been reported in Puerto Rico. In the current study, the proportion of *BRCA* carriers in unselected breast cancers cases was 2.9% (CI 1.5–5.5%). This prevalence increased to 5.2% (2.4–10.8%) in cases diagnosed at or before 50 years of age, and to 5.6% (CI 2.8–11.2%) in cases that met the NCCN criteria for *BRCA* testing [14]. The totality of the patients identified carried a pathogenic variant in *BRCA2*, with the *BRCA2* E1308X truncating variant accounting for 89% of carriers.

Our data clearly establish the *BRCA2* E1308X mutation as a common recurrent founder allele in Puerto Rico. Founder effects can occur when a colony is established from a small subset of individuals originating from a larger population. The genetic makeup of the Puerto Rico population is determined by its early colonial history where the Spanish, African and Taino peoples intermingled for several generations [23]. Previous studies have shown the occurrence of West Eurasian and North African founder haplotypes in Puerto Rico that are informative for specific ethnic groups that populated the island during colonization, and their frequencies have been shaped by random genetic drift [24]. The *BRCA2* E1308X mutation was originally described in two independent families in Spain, each with three or more breast cancer cases, but without ovarian cancer. Duran et al. identified E1308X in a patient from Castilla-Leon, Spain, with an onset at 46 years of age, and three or more affected relatives [25]. In 410 Spanish breast cancer families and 214 isolated cases, *BRCA2* E1308X was observed only once in a family with four breast cancer cases before age 50 and one after 50 [26]. Therefore, *BRCA2* E1308X is present in Spain, but is not a common allele. Our haplotype analysis of *BRCA2* in E1308X carriers strongly supports a common origin, with the carriers sharing a haplotype in a region spanning at least 1.74 megabases around the gene. Consistent with the known origin of this mutation, local ancestry estimates show that all carriers examined shared at least one chromosome copy of European origin in the genomic region where *BRCA2* is located. Interestingly, this variant was identified in patients residing in municipalities from the South of Puerto Rico (Peñuela, Ponce), the center of the island (Cidra, Naranjito), and in the Metropolitan area (Bayamon, San Juan), suggesting that the distribution of this variant within Puerto Rico is not restricted to a specific geographic area. This mutation was previously seen four times in 23 Puerto Rican high-risk subjects [13], and in a subject of Puerto Rican descent living in mainland US [27], and in US Hispanic/Latinos of unknown origin [28,29]. The combined data for Puerto Rico shows that this deleterious variant accounts for over 60% of the identified *BRCA1*/*2* carriers. 

Founder mutations in the *BRCA1*/*BRCA2* genes have first been described in the Ashkenazi Jewish population, with *BRCA1* 185delAG, *BRCA1* 5382insC and *BRCA2* 6174delT representing 79% of the *BRCA1*/*BRCA2* mutations found in this population [30]. Various founder mutations have also been reported in Latin American populations. The Ashkenazi founder mutations are recurrent in Argentina, in a population of known Jewish origin [31]. In Mexico, germline mutations 2805_2808delAGAT and 3124_3133delAGCAATATTA in *BRCA1*, and 2639_2640delTG and 5114_5117delTAAA in *BRCA2* are reported to be deleterious founder mutations [32]. In Colombia, carriers of the 3450del4 and A1708E in *BRCA1* and 3034delACAA in *BRCA2* shared common ancestors [33]. In Brazil, two mutations, 5382insC in *BRCA1*, 6633del5 and 156_157insAlu in *BRCA2* are the most frequent [34]. This Alu insertion mutation has been identified in Portuguese families, suggesting a founder event of this mutation from Portuguese settlers in Brazil [35]. While some of these recurrent mutations have been observed in more than one country, the majority shows limited overlap [12]. Such observations confirm that populations from Latin America and the Caribbean are heterogeneous, resulting from unique combinations of ancestral genetic factors and historical events that have shaped the genetics of those populations today. In a context where access to genetic testing may be limited by economical resources and healthcare infrastructure, in some countries of Latin America, stepwise screening of recurrent mutations followed by complete assessment of *BRCA1* and *BRCA2* in negative cases has been proposed as a cost-effective strategy [36]. Nonetheless, available data indicate that if possible at all, such an approach should take into account the genetic diversity of the populations commonly referred to as Hispanic or Latinos. 

As a result of the increased availability to genetic testing for hereditary cancers, and improved models for functional assessment [37], the proportion of unclassified variants in *BRCA1*/*BRCA2* has been progressively decreasing over time [29]. In this study, 6.8% (*n* = 21) of the tested women were found to carry a missense variant of uncertain significance in *BRCA1* or *BRCA2*, which is a rate lower than what has been previously reported for US Hispanics [29]. Yet, the clinical management of those variants presents a challenge. It has been shown to yield to over- or under- treatment [38], is of limited clinical utility for pre-symptomatic relatives, and can be associated with increased psychological distress [39]. While the clinical relevance of truncating frameshift and nonsense variants in the *BRCA1*/*2* genes is well understood, the classification of missense variants still represents a difficult task. Our recent work in a Puerto Rican cohort provided evidence against pathogenicity of the *BRCA2* c.6937+594T>G variant, therefore emphasizing the benefit to include diverse populations in examining evidence for variant classification [40]. 

For most of the cases that met the NCCN guidelines for *BRCA*-genetic testing, no pathogenic *BRCA* variant was identified. Among those probands, some had family history strongly suggesting a genetic contribution such as early onset and multiple cases of BC, as well as ovarian cancer. In the recent past, testing of panels of susceptibility genes for hereditary cancer has replaced *BRCA1*/*2* testing. Pathogenic variants in *PTEN*, *TP53*, *CHEK2*, *ATM*, *NBS1*, *RAD50*, *BRIP1* and *PALB2*, amongst others, have also been shown to confer moderate to high risk of breast cancer [41,42]. Genome-wide association studies (GWAS) have identified over a hundred loci associated with breast cancer risk, accounting for up to 12% of the familial risk attributed to common variants. Although the risk associated with individual loci is not elevated enough to inform clinical decisions, polygenic risk scores were proposed as risk stratification tools in population screening programs and targeted prevention [43]. Therefore, *BRCA1*/*2* testing is no longer sufficient to rule out the contribution of genetic factors to the cancers and it is possible that some of the patients for which no *BRCA* mutation was identified are carriers of a deleterious variant in another susceptibility gene or genes. Our preliminary work indicates that at least 8.3% of the high-risk BC patients for which no *BRCA* mutation is identified carry a pathogenic variant in another susceptibility gene [44]. 

Compared to our previous work in Puerto Rico, this study presents the advantage of screening for *BRCA* mutations in a larger sample of BC patients that have not been selected for family history or age of onset. It therefore presents more accurate estimates of the proportion of BC cases attributed to *BRCA* pathogenic variants. However, some limitations do remain. Given the sample size, it is unlikely that all the *BRCA* mutations underlying the breast and ovarian hereditary cancer syndrome in this population have been identified. In addition, a sampling is expected as a consequence of the recruitment design through clinics rather than the use of a census-based approach. The cohorts studied represent 58 out of the 78 municipalities of Puerto Rico, but some regions of the island are oversampled due to the proximity of the participating clinics. This is especially the case for the South of the Island, which was overrepresented in relation to population concentration in this area. There was also a lack of patients coming from the Western areas of Puerto Rico where one major urban center is located. Geographic variation in the ancestry proportions of the Puerto Rico population has been previously observed [23]. As a consequence, it is possible that a pathogenic *BRCA* variant that would be more common within a restricted geographic area of Puerto Rico was not detected as a consequence of the sampling approach. 

## 4. Materials and Methods 

### 4.1. Study Population

The study population consists of 232 BC patients recruited through the Ponce Research Institute (PRI) of Ponce Health Sciences University, and 75 patients from the University of Puerto Rico (UPR) Comprehensive Cancer Center. Patients undergoing BRCA analysis and recruited through the PRI are a subset of a larger cohort, which has been described previously [45]. In brief, between 2006 and 2012, in participating clinics, patients were approached by the research nurse or study coordinator and offered to participate in the study. Patients were recruited at the moment of initial diagnosis, were not selected for family history and were diagnosed at any age. Patient recruitment and counseling was approved by IRBs at the Ponce Research Institute (IRB#070918-JD), the University of Puerto Rico Medical Sciences Campus (IRB#A1810111), and under an exemption from the National Institutes of Health (NIH) Office of Human Subjects Protection (Exemption #5252AA) for coded samples sent to the National Cancer Institute (NCI) for sequencing. Cases were identified in oncology and surgery clinics located in the Metropolitan area and the South of the Island but who serve patients from all over the island. Inclusion criteria for cases were patients who (1) were 21 years or older, and (2) were recently diagnosed histopathologically with primary BC. Cases that had been previously treated for cancer at another site were excluded from this protocol. Participants completed a standard questionnaire regarding their demographic information, hormonal and pregnancy history and family history of cancer and a blood sample was drawn for DNA extraction. Additionally, a tumor pathology report was obtained. DNA from three known BRCA pathogenic carriers was included as positive controls for sequencing, two of which were provided by the Corriell Cell Repository (NA13715 *BRCA1* 5382insC, NA14638 *BRCA1* IVS5-11T>G) and from the Ponce Research Institute (*BRCA2* del exon 1-2). 

### 4.2. Sample Preparation and BRCA1/2 Sequencing

Genomic DNA was extracted from blood lymphocytes or whole blood using QIAamp and Paxgene DNA kits (QIAGEN, Germantown, MD USA), respectively. The concentration of DNA was measured using the Nano Drop 2000 spectrophotometer (Thermo Fisher Scientific, Waltham, MA, USA). Libraries were prepared using the Ion Ampliseq Library kit 2.0 (Life Technologies, Calrlsbad, CA, USA) according to the manufacturer’s protocol using 15 ng genomic DNA. Multiplex PCR was performed with Ion AmpliSeq *BRCA1* and *BRCA2* Community Panel (Life Technologies), which consists of 163 primer pairs in three pools and was designed to capture all *BRCA1*/*BRCA2* coding exons and exon-intron junctions (24,143 bp). Following adaptor and barcode ligation, libraries were pooled and amplified by emulsion PCR using the OneTouch2 system and Ion Xpress template kit (Life Technologies). Ion Sphere particles were enriched using the E/S module and sequenced on PGM using a 316 chip (Life Technologies). Full screening of *BRCA1* for the detection of large rearrangements using multiplex ligation-dependent probe amplification (MLPA) was performed according to the manufacturer’s protocol (MRC Holland, Amsterdam, The Netherlands).

### 4.3. Variant, Filtering and Annotation

The sequence data were processed using standard Ion Torrent Suite Software with standard parameters [27]. Variants passing quality controls and filtering were visually confirmed using the Integrative Genomics Viewer (IGV) [46]. Rare variants (with a reported frequency <1% or absent from the 1000 Genomes Puerto Ricans from Puerto Rico (PUR)) were classified according to their position in the gene and annotated using ClinVar [15]. Frequencies from the Exome Aggregation Consortium (ExAC) were also reported [16]. For missense variants, predictions of pathogenicity were generated using Align-GVGD [17], Breast Cancer Gene Prior Probabilities of the Huntsman Cancer Institute (HCI BrCa) [18], Polyphen2 [19], SIFT [20] and Provean [21,22]. 

### 4.4. Ancestry Analyses

Global ancestry proportions of each participant were estimated using a panel of 106 ancestry informative SNPs that can discriminate indigenous American, African, and European ancestry, and distributed across all 22 autosomal chromosomes. Genotyping of the 106 ancestry informative markers for all samples was done by using a multiplex PCR using a Sequenom analyzer. The SNP panel, primers and reaction conditions have been previously described [47]. For each individual, respective proportions of European, African and Native American ancestry were estimated using ADMIXTURE [48]. In pathogenic variant carriers, local ancestry at the site of the genes of interest was extracted from whole genome local ancestry estimates (available for 207 samples). These local ancestry estimates were obtained from a subset 67,000 SNPs genotyped on an Affymetrix Axiom UK Biobank Array. Genotypes were phased using Shape-IT [49] and locus-specific ancestry was determined by RFMix [50]. 

### 4.5. Haplotype Analysis

In genomic regions around recurrent pathogenic variants, haplotypes were determined using a subset of markers from the 67,000 SNPs genotyped on an Affymetrix Axiom UK Biobank Array (73 SNPS in a 2.25 Megabases (Mb) genomic interval around BRCA2, GRCh37/hg19 chr13:31.92–34.16 Mb) and phased using PHASE v2.1.1 [51] using the –x5 flag. 

### 4.6. Statistical Analysis

All statistical analyses were conducted with R version 3.2.1 implemented in R Studio [52]. Differences in frequency distributions were calculated by Pearson Chi-square χ^2^ test. 

## 5. Conclusions

We have demonstrated that in unselected BC cases from Puerto Rico, the prevalence of the *BRCA1* and *BRCA2* mutations is 2.9%. BRCA2 is the predominant gene mutated in breast cancer patients in this population, with a single mutation, E1308X accounting for 88% of the alleles. Further screening of this mutation could aid in the early diagnosis prevention and reduction of mortality of breast cancer.

## Figures and Tables

**Table 1:** *BRCA1* and *BRCA2* pathogenic variants detected in Puerto Rican breast cancer patients.

Gene/Exon	AA	HGVS	No	dbSNP	ExAC Frequencies ^3^			
	Change	Positions ^1^	obs. ^2^		Overall	Eur	Afr	Lat
*BRCA1*								
Exon 6	Stop 75	c.213-11T>G (g.41256984A>C)	*	rs80358061	8.3 × 10^−6^	1.5 × 10^−5^	0	0
Exon 20	Stop 1829	c.5263_5264insC (g.41209082_41209083insG)	*	rs80357906	1.6 × 10^−4^	2.8 × 10^−4^	0	0
*BRCA2*								
Exon 10	Stop 599	c.1794_1798delATTTT (g.32907409_32907413delATCTT)	1	NA	NA	NA	NA	NA
Exon 11	E1308X	c.3922G>T (g.32912414G>T)	8	rs80358638	NA	NA	NA	NA

^1^ Refers to position on genome assembly hg19/GRCh37. ^2^ Number of observations in the current study. ^3^ Frequencies reported by the Exome Aggregation Consortium (ExAC) populations [15]. Afr African, Eur European (non-Finnish), Lat Latino, HVGS Human Genome Variation Society, NA not available. * Positive samples from Coriell Biorepository.

**Table 2:** *BRCA1* and *BRCA2* missense variants of uncertain significance detected in Puerto Rican breast cancer patients.

Gene/Exon	AA	HGVS	No	dbSNP	ExAC Frequencies ^3^			
	Change	Positions ^1^	obs. ^2^		Overall	Eur	Afr	Lat
*BRCA1*								
Exon 11	I571T	c.1712C>T (g.41245836A>G)	3	rs80357159	1.7 × 10^−5^	0	0	1.7 × 10^−4^
Exon 11	F1231L	c.3691T>C (g.41243857A>G)	1	rs41293451	3.3 × 10^−5^	0	3.8 × 10^−4^	0
Exon 11	I1275V	c.3823A>G (g.41243725T>C)	1	rs80357280	1.1 × 10^−4^	7.5 × 10^−5^	0	7.8 × 10^−4^
Exon 13	H1421R	c.4262A>G (g.41234516T>C)	1	rs80357079	NA	NA	NA	NA
Exon 13	H1421Y	c.4261C>T (g.41234517G>A)	1	rs80357013	1.6 × 10^−5^	0	9.6 × 10^−5^	8.6 × 10^−5^
Exon 14	E1470D	c.4410A>T (g.41228579T>A)	4	rs80357075	2.5 × 10^−5^	0	0	2.6 × 10^−4^
*BRCA2*								
Exon 10	I283V	c.847A>G (g.32906462A>G)	1	rs80359097	NA	NA	NA	NA
Exon 10	I488V	c.1462A>G (g.32907077A>G)	1	NA	NA	NA	NA	NA
Exon 10	Y600H	c.1798T>C (g.32907413T>C)	1	rs75419644	4.9 × 10^−4^	0	5.8 × 10^−3^	1.7 × 10^−4^
Exon 11	K1058R	c.3173A>G (g.32911665A>G)	1	rs431825302	1.7 × 10^−5^	0	0	1.7 × 10^−4^
Exon 11	D1923A	C.5768A>C (g.32914260A>C)	1	rs45491005	2.8 × 10^−4^	0	3.2 × 10^−3^	8.6 × 10^−5^
Exon 11	Q2159E	c.6475C>G (g.32914967C>G)	2	NA	NA	NA	NA	NA
Exon 22	K2950N	c.8850G>T (g.32953549G>T)	1	rs28897754	6.8 × 10^−4^	8.1 × 10^−4^	9.8 × 10^−5^	1.8 × 10^−3^
Exon 27	P3292L	c.9875C>T (g.32972525C>T)	1	rs56121817	7.4 × 10^−5^	4.5 × 10^−5^	0	8.7 × 10^−5^

^1^ Refers to position on genome assembly hg19/ GRCh37. ^2^ Number of observations in the current study. ^3^ Frequencies reported by the Exome Aggregation Consortium (ExAC) populations [16]. Afr African, Eur European (non-Finnish), Lat Latino, HVGS Human Genome Variation Society, NA not available.

**Table 3:** In silico predictions of functionality for *BRCA1* and *BRCA2* missense variants of uncertain significance.

Gene/Variant	ClinVar ^1^	Align GVGD ^2^	HCI Probability Pathogenicity		Polyphen ^2^	SIFT ^3^	Provean ^4^
			Protein	*de novo* Donor Site			
*BRCA1*							
I571T	VUS	C0	Weak/Null	Weak/Null	Benign	Tolerated	Neutral
F1231L	Conflicting	C0	Weak/Null	Weak/Null	Possibly damaging	Damaging	Neutral
I1275V	Conflicting	C0	Weak/Null	Increased	Benign	Tolerated	Neutral
H1421R	Conflicting	C15	Weak/Null	Weak/Null	Possibly damaging	Damaging	Neutral
H1421Y	VUS	C0	Weak/Null	Weak/Null	Benign	Tolerated	Neutral
E1470D	Conflicting	C0	Weak/Null	Weak/Null	Benign	Damaging	Neutral
*BRCA2*							
I283V	Conflicting	C0	Weak/Null	Weak/Null	Benign	Tolerated	Neutral
I488V	VUS	C0	Weak/Null	Weak/Null	Benign	Tolerated	Neutral
Y600H	Conflicting	C0	Weak/Null	Weak/Null	Benign	Tolerated	Neutral
K1058R	Conflicting	C0	Weak/Null	Weak/Null	Benign	Tolerated	Neutral
D1923A	Conflicting	C0	Weak/Null	Weak/Null	Benign	Damaging	Deleterious
Q2159E	Conflicting	C0	Weak/Null	Weak/Null	Benign	Tolerated	Neutral
K2950N	Conflicting	C35	Moderate	Weak/Null	Probably damaging	Damaging	Neutral
P3292L	Conflicting	C0	Weak/Null	Weak/Null	Probably damaging	Damaging	Deleterious

^1^ Conflicting ClinVar classification refers to variants for which there were contradicting classifications as benign or uncertain significance depending on the source of the clinical report. ^2^ Align GVGD grades were retrieved from the Huntsman Cancer Institute, University of Utah. ^3^ Cutoff for the classification of a variant as damaging was a score < 0.05. ^4^ Cutoff for the classification of a variant as deleterious was a score < −2.5. NA not available, VUS variant of uncertain significance, HCI Huntsman Cancer Institute.

**Table 4:** Tumor pathology and family history characteristics of the deleterious *BRCA* variant carriers.

Gene/Variant Patient Identification	NCCN ^1^	Age dx	Tumor Type	Tumor Size (cm)	Lymph Nodes	Receptors	Family History of Cancers
*BRCA2* c.1794_1798del5							
UPR1024	NA	52	NA	2.0	negative	PR^−^, HER2^-^	Sister, breast (dx NA)
*BRCA2* E1308X							
PRI1154	yes	55	ductal, in situ	NA	negative	ER^+^, PR^+^	Maternal uncle, breast (dx 60 yrs)
PRI1304	yes	33	NA	NA	NA	NA	Sister, breast (dx 46); sister, breast (dx 47); father, liver (dx 77)
PRI1657	yes	24	ductal, invasive	NA	NA	ER^+^, PR^+^, HER2^-^	Paternal grand-parent, gastric (dx 83)
PRI1699	yes	50	ductal, in situ	1.5	NA	ER^+^, PR^+^	Mother, ovarian (dx 69); paternal uncle, breast (dx 62)
PRI1713	yes	38	lobular, invasive	1.5	negative	ER^+^, PR^+^	Maternal aunt, breast (dx 40)
PRI1936	yes	46	ductal, in situ	NA	NA	ER^+^, PR^+^, HER2^-^	Father, pancreas (dx 50); paternal aunt, breast (dx 60); maternal uncle, breast (dx 54); maternal grand-mother, breast (dx 75)
PRI1949	NA	53	ductal, in situ	0.1	NA	ER^+^, PR^+^, HER2^+^	Father, prostate (dx 70); brother, prostate (dx 47), sister, thyroid (dx 58)
UPR1043	yes	40	NA	2.1	negative	ER^+^, PR^+^, HER2^+^	Sister, breast (dx NA)

^1^ NCCN version 2.2017. Dx age of diagnosis (years), ER estrogen receptor, PR progesterone receptor, HER2 human epithelial growth factor receptor, NA not available or unable to determine.

## Supplementary Materials

The following are available online at http://www.mdpi.com/2072-6694/10/11/419/s1, Table S1: demographic, reproductive and hormonal characteristics of the study population; Table S2: breast tumor pathology and family history characteristics of the study population; Table S3: *BRCA1* and *BRCA2* intronic variants of uncertain significance; Figure S1: geographic distribution of the study population; Figure S2: global ancestry proportion of the study population and local ancestry at the *BRCA2* locus in carriers of the E1308X mutation.
