# Supplementary material for: A Recurrent BRCA2 Mutation Explains the Majority of Hereditary Breast and Ovarian Cancer Syndrome Cases in Puerto Rico
Source: Cancers (Basel). 2018 Nov 2;10(11):419. doi: 10.3390/cancers10110419 (PMC6266560; doi:10.3390/cancers10110419)
Supplement: Supplementary file 1 [file cancers-10-00419-s001.pdf]

Supplementary Materials: A Recurrent *BRCA2* Mutation Explains the Majority of Hereditary Breast and Ovarian Cancer Syndrome Cases in Puerto Rico

Hector J. Diaz-Zabala, Ana P. Ortiz, Lisa Garland, Kristine Jones, Cynthia M. Perez, Edna Mora, Nelly Arroyo, Taras K. Oleksyk, Miguel Echenique, Jaime L. Matta, Michael Dean and **Julie Dutil**

Table S1. Demographic, reproductive and hormonal characteristics of the study population.

Variable		% (*n*)
Demographic		
Age		
	<40 yrs	10.8 (32)
	40–49	22.7 (67)
	50–59	31.2 (92)
	60–69	24.4 (72)
	≥70	10.8 (32)
BMI		
	<25	28.2 (83)
	25–29	38.1 (112)
	≥30	33.7 (99)
Civil status		
	Single	20.0 (59)
	Married/Union	53.2 (157)
	Divorced/Separated	15.3 (45)
	Widow	11.5 (34)
Education level		
	Up to high school	40.2 (103)
	Associate	22.7 (58)
	Bachelor	37.1 (95)
Hormonal & pregnancy history		
Age at menarche		
	≥13	43.1 (125)
	<13	56.9 (165)
Oophorectomy		
	None or unilateral	83.2 (242)
	Bilateral	16.8 (49)
Oral contraceptive		
	Never used	45.2 (131)
	Ever used	54.8 (159)
Pregnancy		
	Ever been pregnant	88.8 (261)
	Never been pregnant	11.2 (33)

Number of children	
Nulliparous	14.7 (40)
1–2	44.7 (131)
≥3	41.6 (122)
Menopause [1]	
Premenopausal	32.8 (95)
Peri-/post-menopausal	67.2 (195)
Estrogen replacement therapy	
Ever used	16.7 (44)
Never used	83.3 (219)

[1] Peri-/post-menopause includes natural and induced menopause. The total number of subjects varies across variables because of missing values (n missing was age 12, BMI 13, civil status 12, education level 51, age at menarche 17, oophorectomy 16, oral contraceptives 17, pregnancy 13, number of children 14, menopause 17, estrogen replacement therapy 44. BMI body mass index.

Table S2. Breast tumor pathology and family history characteristics of the study population.

Variable	% (*n*)
Breast tumor pathology	
Age at diagnosis	
≤50	39.7 (116)
>50	60.3 (176)
Site/type	
Ductal carc. in situ	21.5 (55)
Ductal invasive carc.	71.1 (182)
Lobular invasive carc.	7.4 (19)
Size (centimeters)	
≤2	64.4 (121)
>2	35.6 (67)
Lymph nodes	
Negative	67.7 (105)
Positive	32.3 (50)
ER	
Negative	36.1 (83)
Positive	63.9 (147)
PR	
Negative	38.9 (68)
Positive	71.1 (167)
HER2	
Negative	66.8 (135)
Positive	33.2 (67)
Family history [1]	
Breast cancer	
None	69.6 (204)
At least one relative	30.4 (89)

Male breast cancer	
None	98.3 (288)
At least one relative	1.7 (5)
Ovarian cancer	
None	96.9 (284)
At least one relative	3.1 (9)
Other BRCA-associated cancers [2]	
None	81.2 (238)
At least one relative	18.8 (55)
Other cancers	
None	47.1 (138)
At least one relative	52.9 (155)
Meet NCCN criteria for BRCA genetic testing [3]	
Yes	45.9 (124)
No	54.1 (146)

[1] Includes first-, second- and third- degree relatives on paternal and maternal side. [2] Other *BRCA*-associated cancers included pancreatic cancer, prostate cancer and melanoma. [3] Version2.2017. The total number of subjects varies across variables because of missing values (n missing was age at diagnosis 15, site/type 51, size 119, lymph nodes 152, ER 77, PR 72, HER2 105, family history 14, NCCN criteria 37. Carc. carcinoma, ER estrogen receptor, progesterone receptor PR, human epithelial growth factor HER2, NCCN National Comprehensive Cancer Network.

Table S3. *BRCA1* and *BRCA2* intronic variants of uncertain significance.

Gene	Exon	HGVS cDNA	HGVS_Genomic [1]	No obs. [2]	dbSNP ID	ExAC Frequencies				ClinVar [3]
						Overall	Eur	Afr	Lat	
BRCA1										
	7	c.441 + 41C>T	g.41256098G>A	7	rs45489593	$2.4x10^{-04}$	$2.4x10^{-04}$	0	$3.8x10^{-04}$	VUS
	7	c.441 + 52T>C/ c.441 + 51C>T	g.41256087_ 41256088delinsAG	14	NA	NA	NA	NA	NA	NA
	9	c.548-9_548-9delA	g.41249315delT	1	rs273902774	NA	NA	NA	NA	Conflicting
	19	c.5312 + 22C>T	g.41215328G>A	1	rs8176260	$7.6x10^{-05}$	0	$8.9x10^{-04}$	0	VUS
BRCA2										
	2	c.-15A>C	g.32890583A>C	1	rs138705202	$2.2x10^{-04}$	0	$2.2x10^{-03}$	$2.6x10^{-04}$	Conflicting
	3	c.68-7insA	g.32893207insA	1	NA	$2.4x10^{-03}$	$5.5x10^{-03}$	$3.2x10^{-04}$	$1.0x10^{-03}$	NA
	10	c.1909 + 141A>G	g.32907673A>G	1	NA	NA	NA	NA	NA	NA
	11	c.6841 + 55T>G	g.32915388	1	NA	NA	NA	NA	NA	NA
	13	c.7007 + 53G>A	g.32921086	1	rs56014558	NA	NA	NA	NA	NA
	24	c.9256 + 58A>T	g.32954340A>T	1	NA	NA	NA	NA	NA	NA
	26	c.9502-40T>A	g.32970995T>A	1	NA	NA	NA	NA	NA	NA

[1] Refers to position on genome assembly hg19/ GRCh37. [2] Number of observations in the current study. [3] Conflicting ClinVar classification refers to variants for which there were contradictory classifications as benign or uncertain significance depending on the source of the clinical report. Afr African, Eur European, Lat Latino, VUS variant of uncertain significan.

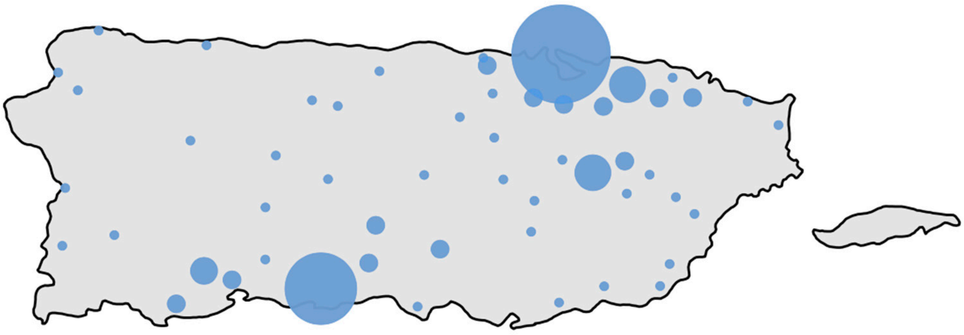

(**a**)

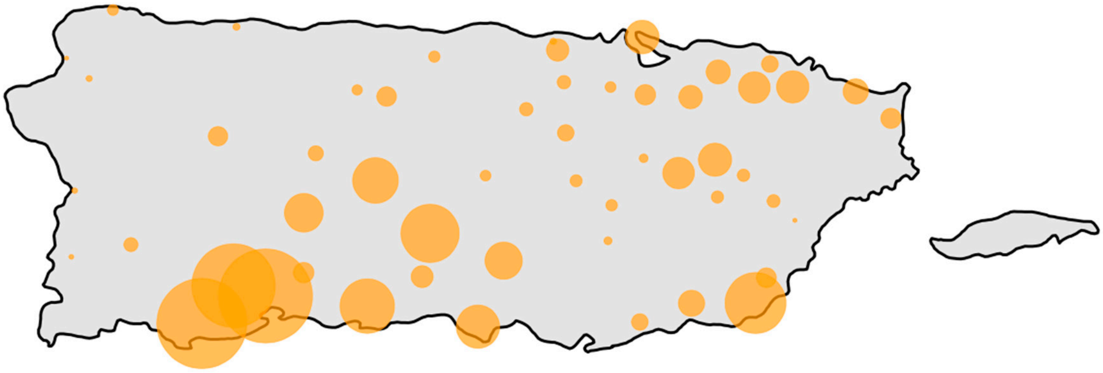

(**b**)

Figure S1. Geographic distribution of the study population. Measured by municipality of residence, and expressed as (**a**) absolute counts and (**b**) relative to population density.

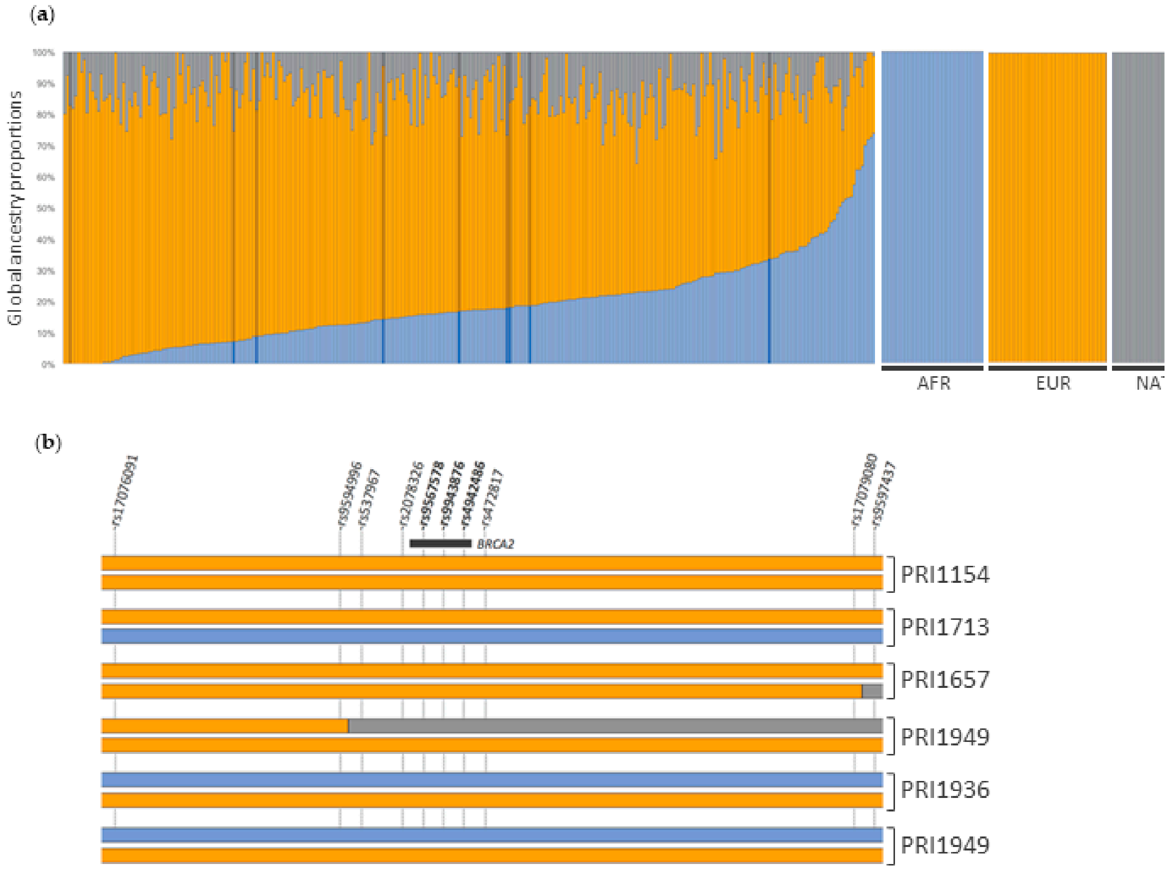

Figure S2. Global ancestry proportions in the study population and local ancestry at the *BRCA2* locus in E1308X mutation carriers. Global ancestry proportion (**a**) in study population in comparison to reference ancestral populations (African AFR, European EUR, and Native American NAT). *BRCA* pathogenic mutation carriers are illustrated by darker bars. Local ancestry estimates at the *BRCA2* locus in E1308X carriers (**b**). In B, only six of the eight *BRCA2* E1308X carriers are represented, which corresponds to the samples for which genome-wide SNP data was available.
